# A Case of Bilateral Trochlear Nerve Palsy Induced by a Dorsal Midbrain Intracranial Lipoma

**DOI:** 10.7759/cureus.95704

**Published:** 2025-10-29

**Authors:** Junya Kawamura, Yukiko Shimizu, Hitoshi Tabuchi

**Affiliations:** 1 Department of Ophthalmology, Saneikai Tsukazaki Hospital, Himeji, JPN

**Keywords:** bilateral trochlear nerve palsy, dorsal midbrain, excyclotropia, intracranial lipoma, strabismus surgery

## Abstract

Intracranial lipomas are rare among all intracranial tumors. They are typically asymptomatic and discovered incidentally through neuroimaging. However, in rare cases, these lesions can produce neurological symptoms by compressing adjacent structures. We report a rare case of bilateral trochlear nerve palsy caused by an intracranial lipoma located in the dorsal midbrain. A 67-year-old man presented with long-standing binocular diplopia and visual distortion, particularly a perception of tilted vertical lines. Neuro-ophthalmological examination revealed bilateral trochlear nerve dysfunction, with significant excyclotropia and vertical deviation, more pronounced on the left side. Magnetic resonance imaging demonstrated a high-intensity lesion on both T1- and T2-weighted sequences, consistent with a lipoma in the dorsal midbrain. Given the lesion's benign nature and deep location, surgical resection was not indicated due to the high risk of neurological complications. The patient underwent bilateral nasal transposition of the inferior rectus muscles, with additional recession on the right side, to correct the torsional and vertical misalignment. Postoperative assessment demonstrated marked improvement in ocular alignment, restoration of stereopsis, and complete resolution of diplopia. This case underscores the importance of considering intracranial lipoma in the differential diagnosis of bilateral trochlear nerve palsy, especially in patients without a history of trauma. Although surgical removal of the lesion was not feasible, targeted strabismus surgery successfully alleviated the patient’s symptoms. However, since the tumor has not been removed, long-term follow-up is essential to monitor for any progression of neurological symptoms due to the underlying lesion.

## Introduction

Lipomas are benign tumors that rarely occur intracranially. Intracranial lipomas were first described by Meckel in 1818 and have since been reported in various papers, with a frequency of less than 1% of all intracranial tumor cases [1-3]. They are usually asymptomatic and are often discovered incidentally on imaging tests for the diagnosis of other diseases and symptoms [4,5]. However, it should be noted that it can also present with symptoms such as epilepsy, persistent headache, seizures, mental retardation, and cranial nerve palsy [6]. The diagnosis of intracranial lipoma is confirmed by pathological examination of tissue taken by biopsy. However, since it may involve surrounding blood vessels and nerves and may cause bleeding and neurological symptoms during removal, surgery is often not performed, and the patient is followed up with a clinical diagnosis even if detected by imaging tests [7].

The trochlear nucleus is located caudal to the oculomotor nucleus of the midbrain. From this nucleus, nerve bundles arise, which proceed posteriorly and inferiorly around the midbrain aqueduct and cross in the anterior medullary velum just caudal to the inferior colliculus. The trochlear nerve emerges as one or more nerve rootlets from the dorsal surface of the lower midbrain just below the inferior colliculus, near the tentorium. The axons of the trochlear nerve leave the midbrain contralateral to their nucleus of origin [8].

The trochlear nerve innervates the superior oblique muscle, whose primary function is intorsion of the eye, with additional actions including downward and slight abduction [8]. Therefore, trochlear nerve palsy presents with extorsion and vertical strabismus in unilateral cases, while bilateral involvement results in pronounced extorsional strabismus [9].

When an intracranial tumor involves the decussation of the trochlear nerves, it results in bilateral trochlear nerve palsy [10,11]. We report a case of bilateral trochlear nerve palsy caused by a dorsally located midbrain intracranial lipoma, which was incidentally discovered during imaging performed to investigate the cause of strabismus.

## Case presentation

A 67-year-old male patient had noticed a symptom of seeing the center line of the road as if it were slanted since his 50s. Although this symptom had shown no significant worsening over time, he decided he wanted treatment and came to the hospital. The patient wanted treatment for his diplopia in order to pass the stereopsis test required to renew his commercial driver's license. He reported no history of trauma and was being managed medically for hypertension. There were no subjective symptoms other than ocular findings, such as headache.

**Figure 1:**
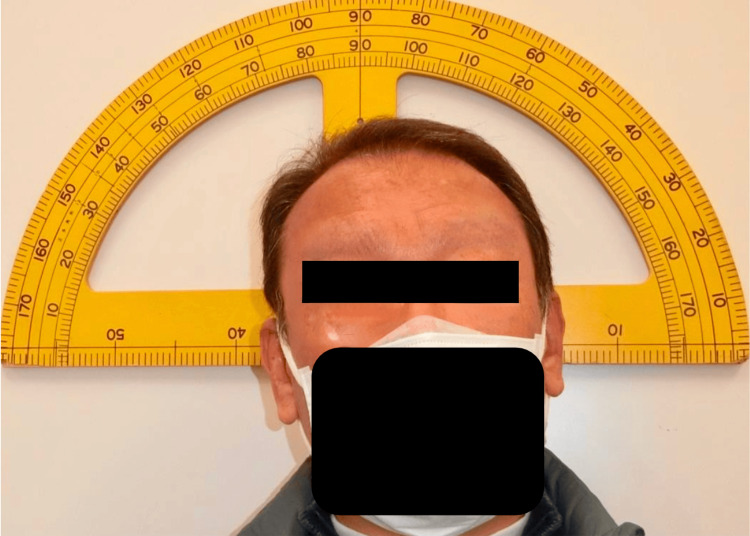
Preoperative natural head position photograph. Right head tilting, chin raising and left face turning are observed.

**Figure 2:**
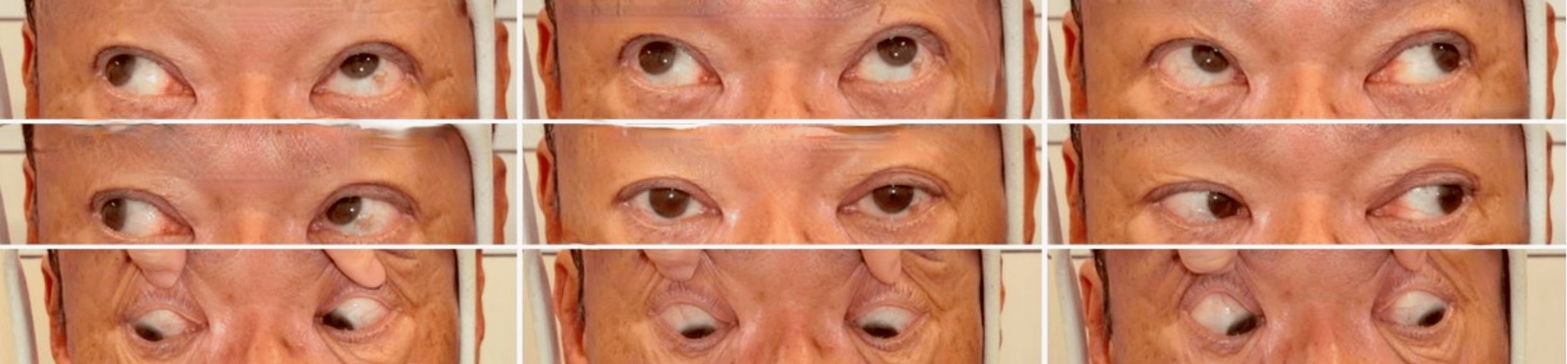
Preoperative photograph of the nine-gaze eye. V-pattern exotropia is observed, with greater exodeviation in upgaze than in downgaze. Overaction of the left inferior oblique muscle is noted as overelevation of the left eye in the right gaze. Both eyes show limitation of movement in the inferomedial direction, more pronounced in the left eye.

**Figure 3:**
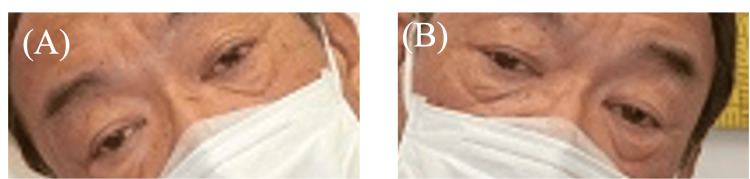
Preoperative head tilt photograph Left hypertropia worsens with left head tilt. (A) Head tilt to right: 16PD of exotropia and 3PD of left hypertropia (by APCT at distance). (B) Head tilt to left: 18PD of exotropia and 20PD of left hypertropia (by APCT at distance)

**Figure 4:**
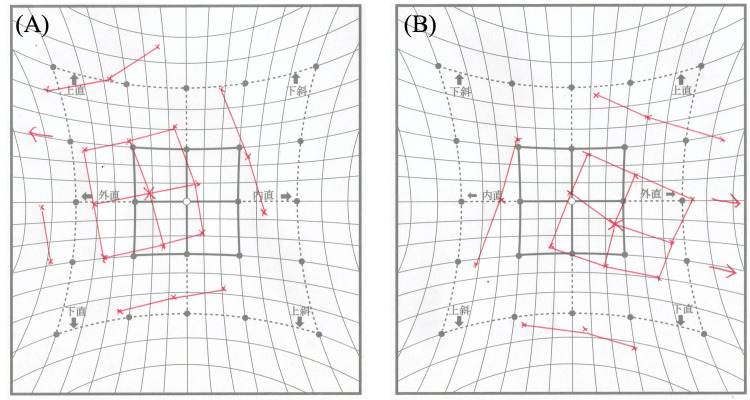
Preoperative Hess chart. (A) Left eye, (B) Right eye Exotropia with left hypertropia. Limitation of inferomedial eye movement is present in the left eye.

**Figure 5:**
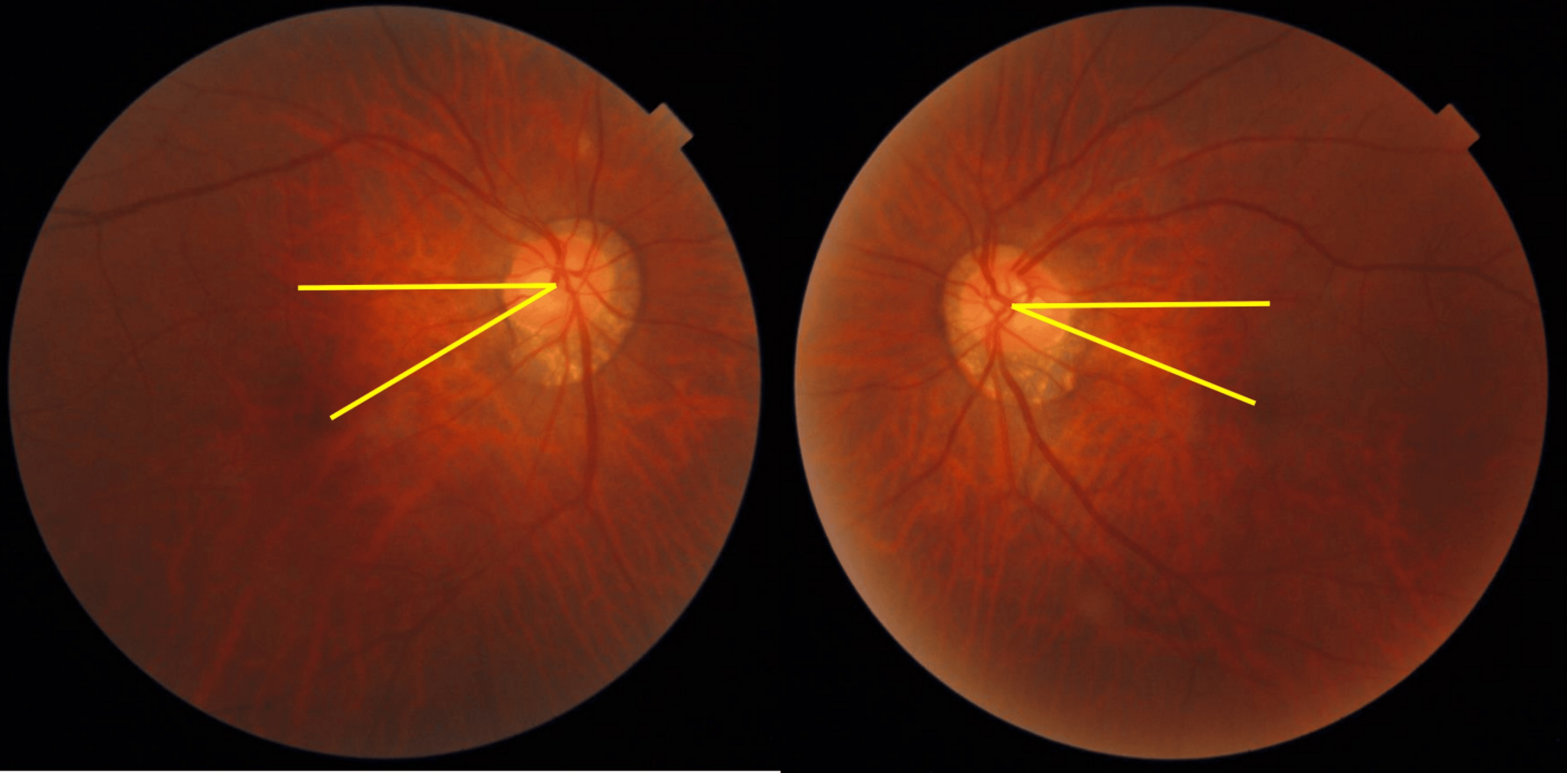
Preoperative fundus photograph A horizontal line drawn from the center of the optic disc and a line drawn from the disc to the macula. Due to extorsion of both eyes, the maculae are displaced inferiorly.

**Figure 6:**
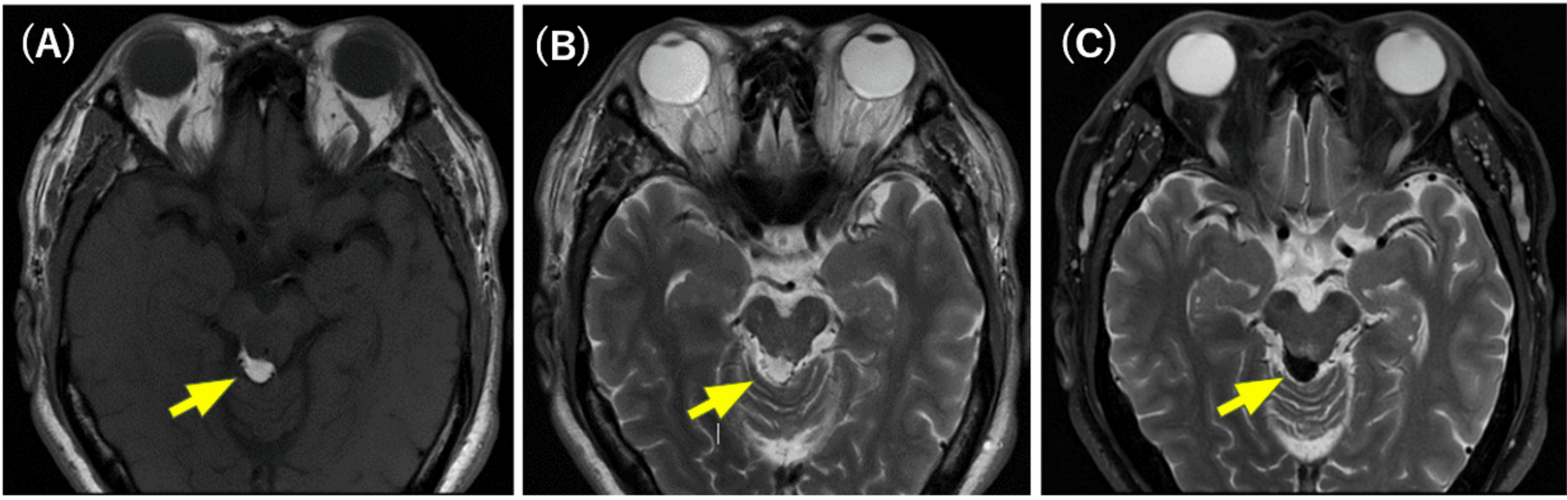
MRI image of the preoperative horizontal section. (A) is a T1-weighted image and (B) is a T2-weighted image, (C) is a fat suppression on T2-weighted image. In (A) and (B) images, a high-signal mass is observed in the area indicated by the arrow, but  in (C) images, a low-signal mass is observed in the area indicated by the arrow.

Upon initial examination, the patient demonstrated a head tilt to the right and limitation of inferomedial gaze in both eyes, more pronounced in the left eye (Figures 1-4). The alternate prism cover test (APCT) revealed 18 prism diopters (PD) of exotropia and 10 PD of left hypertropia in the primary position. With a right head tilt, 16 prism diopters of exotropia and 3 prism diopters of left hypertropia were observed, and with a left head tilt, 18 prism diopters of exotropia and 20 prism diopters of left hypertropia were observed. Stereopsis was absent on the Titmus stereo test (Table 1). His corrected visual acuity was 1.2 in the right eye and 1.2 in the left eye, and anterior segment examination identified cataracts without other abnormalities. Fundoscopic examination noted bilateral extorsion, measured at 25 degrees with a cyclophorometer (Figure 5). Magnetic resonance imaging (MRI) of the brain showed a high-signal-intensity mass in the dorsal midbrain on both T1- and T2-weighted images, diagnosed as a lipoma based on its location and imaging characteristics (Figure 6).

**Table 1:** Preoperative and postoperative test results Measurement in the primary position was performed using the alternate prism cover test. Extorsion was assessed with a cyclophorometer, and stereopsis was evaluated using the Titmus stereo test. PD: prism diopters, XT: exotropia, L/R HT: left hypertropia, Sec: second of arc

	Primary position	Extorsion	Stereopsis
Preoperative	18PD XT 10PD L/R HT	25°	absent
Postoperative	16PD XT 6PD L/R HT	5°	60sec

There was no history of trauma, and the onset was not sudden, as typically seen in vascular cases. MRI revealed a lipoma at the decussation of the trochlear nerves. As there were no signs of thinning or asymmetry of the superior oblique muscles, a congenital etiology was considered unlikely. Given the presence of extorsion in both eyes, a diagnosis of bilateral trochlear nerve palsy caused by an intracranial lipoma. The patient underwent conservative management for the intracranial lipoma as neurosurgical evaluation suggested a high risk of morbidity with surgical intervention due to the lesion's benign nature and deep location. The decision was made to address the symptomatic diplopia with strabismus surgery.

**Figure 7:**
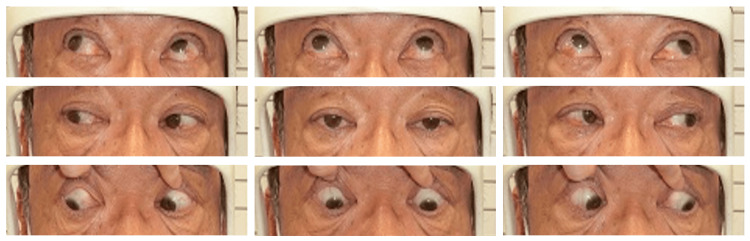
Postoperative photograph of the nine-gaze eye. Improvement of the V pattern exotropia was observed.

**Figure 8:**
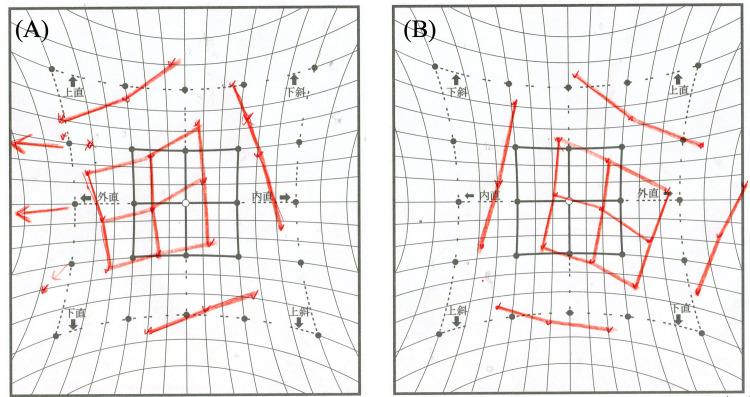
Postoperative Hess chart. (A) Left eye, (B) Right eye Vertical deviation has improved compared to the preoperative findings.

**Figure 9:**
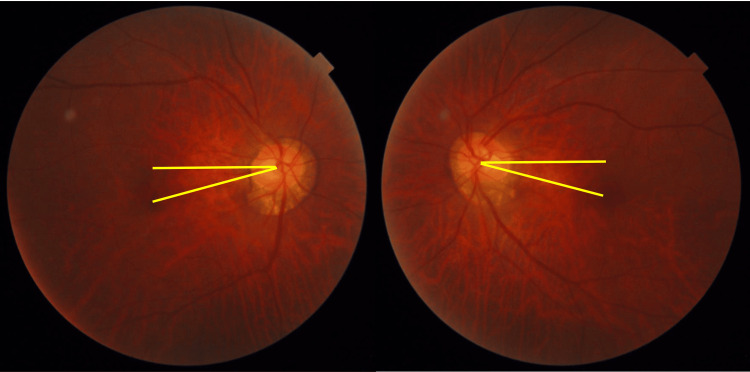
Fundus images at three months postoperatively. Extorsion has improved compared to the preoperative state.

Preoperatively, the patient was fitted with an 8 PD base-up prism for the right eye, which showed exotropia of 18 PD and left hypertropia of 2 PD, with improvement in left hypertropia and disappearance of diplopia. Therefore, treatment was aimed at correcting the left hypertropia and bilateral excyclotropia, and the surgery was planned based on the report by von Noorden et al. [12]. Specifically, in a position along the spiral of Tillaux, surgical correction involved nasal transposition of the inferior rectus muscle on both eyes, with an additional 2 mm of recession on the right eye. At three months postoperatively, APCT of the primary eye position showed 16 PD of exotropia in the distance, 6 PD of left hypertropia, and 5° of extorsion. Extorsion improved by more than 10°, with recovery of stereopsis and disappearance of diplopia (Table 1, Figures 7-9). The patient reported significant improvement in daily visual activities and satisfaction with the surgical outcome.

## Discussion

In this case, we concluded that the cause of the trochlear nerve palsy was a lipoma that appeared in the central part of the dorsal midbrain. Intracranial lipomas are frequently found in the midline or near the midline, such as in the longitudinal fissure around the corpus callosum, the fourth ventricle, the superior cerebellar cistern, the suprasellar cistern, and the interpeduncular cistern [3]. In many cases, they are discovered incidentally and are asymptomatic, but they may present with epilepsy, persistent headaches, seizures, mental developmental delay, and brain nerve paralysis [7]. On MRI, they show high signal on T1- and T2-weighted images and low signal on fat-suppressed images [13]. In this case, the lesion was located in the midline of the dorsal midbrain and demonstrated these characteristic signal patterns, leading to a diagnosis of lipoma. In addition, more than 80% of cases of trochlear nerve palsy are unilateral, and bilateral cases are rare. More than half of bilateral cases are caused by trauma, and other causes include congenital abnormalities, ruptured arteriovenous malformations, and tumors [14-17] (Table 2). The cause of bilateral trochlear nerve palsy due to trauma is damage to the upper medullary falx, but this can also be caused by tumor compression or ischemia. In this case, there was no clear history of trauma or thinning of the superior oblique muscle; a tumor was present that was pressing on the superior medullary velum, and the condition had appeared in the 50s and was gradually progressing, so we considered it to be bilateral trochlear nerve paralysis caused by the compression of a lipoma.

**Table 2:** Origin of trochlear nerve palsy Of the three cases, one was reported as idiopathic, while the remaining two were classified under “other” (tumor, vascular, myasthenia gravis).

	No. of case	Unilateral	Bilateral	Etiology (bilateral)					
				Congenital	Trauma	Presumed microvascular	Undetermined	Tumor	Other
Mollan et al. [14]	150	140	10	1	5		2	2	
Dosunmu et al. [15]	74	70	4	1	3				
Lekskul et al. [16]	158	154	4		3				1
Von Noorden et al. [17]	270	241	29	7	19				3※

Choi et al. prescribed prism glasses for trochlear nerve palsy due to an intracranial lipoma [18]. However, in this case, the angle of extorsion was large, we considered which could not be corrected with prism glasses, so surgery was planned in the same way as for other acquired trochlear nerve palsies. Surgical procedures for acquired trochlear nerve palsy include inferior oblique weakening, the Harada-Ito procedure, and nasal transposition of the inferior rectus muscle. Although the inferior oblique muscle reduction and the Harada-Ito method improve excyclotropia, quantitative changes in postoperative vertical deviation are unstable [19,20]. Therefore, in the present case, we performed recession and nasal transposition of the inferior rectus muscle, which can simultaneously correct external rotation and vertical deviation, based on the report of Von Noorden et al. [12].

There have been a few reported cases similar to this one in which an intracranial lipoma caused trochlear nerve palsy [18]. In this case, there was no history of trauma; it was not sudden, such as vascular; there was a lipoma at the intersection of the trochlear nerve in MRI; and both eyes were extorsion, so we diagnosed bilateral trochlear nerve palsy caused by intracranial lipoma. However, a limitation of this case is that the possibility that the cause of the bilateral trochlear nerve palsy was decompensated bilateral trochlear nerve palsy cannot be ruled out.

## Conclusions

Since trauma is the most common cause of bilateral trochlear nerve palsy, it is essential to obtain a detailed history of head trauma in patients suspected of having this condition. In the absence of a traumatic history, as in the present case, the possibility of a tumor should be considered, and neuroimaging should be performed. Even if the tumor remains within the intracranial adipose tissue, functional improvement through strabismus surgery, as demonstrated in this case, may be a viable option. However, since the tumor has not been resected, careful follow-up is required to monitor for potential progression of symptoms in the future.
